# Follow-up of breast papillary lesion on core needle biopsy: experience in African-American population

**DOI:** 10.1186/1746-1596-9-86

**Published:** 2014-04-24

**Authors:** He Wang, Patricia Tsang, Cyril D’Cruz, Kevin Clarke

**Affiliations:** 1 Department of Pathology and Lab Medicine, Temple University School of Medicine, 3401 North Broad Street, Room 350, Philadelphia, PA 19140, USA; 2 Department of Laboratory Medicine and Pathology, Newark Beth Israel Medical Center, 201 Lyons Avenue, Newark NA 07112, USA; 3 Department of Surgery, Newark Beth Israel Medical Center, 201 Lyons Avenue, Newark NA 07112, USA

**Keywords:** Breast papillary lesion, Core needle biopsy, Immunostains, African-Americans

## Abstract

**Background:**

The optimal course of clinical follow-up after a diagnosis of breast papillary lesion on a core needle biopsy (CNB) remains elusive. In particular, no reports in literature have addressed this question in African-American population. We describe our experience with breast papillary lesions in a primarily African-American population.

**Methods:**

A search of our database for breast papillary lesions diagnosed on CNB between September 2002 and September 2012 was conducted. Cases were categorized into benign, atypical, and malignant. CK5/6 and CK903 stains were performed when necessary.

**Results:**

A total of 64 breast papillary lesions were diagnosed on CNB, including 55 (86%) benign papillary lesions, 6 (9%) atypical lesions, and 3 (5%) intraductal papillary carcinomas. Of these 64 patients, 29 patients (25 African-Americans, 3 Hispanics, 1 Asian American) underwent lumpectomy within 6 months after CNB. Pathology of the lumpectomy showed: five of the 25 (20%) benign papillary lesions on needle biopsy were upgraded to intraductal or invasive papillary carcinoma; 2 of the 3 atypical papillary lesion cases on core biopsy were upgraded (67%), one into intraductal papillary carcinoma, the other invasive papillary carcinoma; the only case of malignant papillary lesion on CNB remained as intraductal papillary carcinoma on lumpectomy. The rate of upgrade in lumpectomy/mastectomy was 25%. CK5/6 and CK903 immunostains were performed on all seven core needle biopsies that were later upgraded.

**Conclusions:**

In our predominantly African-American urban population, 25% of benign or atypical papillary lesions diagnosed on CNB was upgraded in the final excisional examination. Early excision of all papillary lesions diagnosed on CNB may be justified in this patient population.

**Virtual Slides:**

The virtual slide(s) for this article can be found here: http://www.diagnosticpathology.diagnomx.eu/vs/7950117821177201

## Background

In current medical practice, most institutions recommend surgical excision for atypical and malignant papillary breast lesions diagnosed by ultrasound-guided core needle biopsy (CNB). However, both surgical excision and serial radiographic follow-up have been recommended by some authors as appropriate management for benign papillary lesions diagnosed by CNB [1-3]. A major argument for surgical excision of nonmalignant papillary lesion diagnosed by CNB is an unacceptable upgrading rate after excisional specimen evaluation, ranging from 0% to 29% [4,5]. Several clinical and radiological variables, including patient’s age, lesion size, BI-RADS category, and microcalcification, have been examined regarding their predicting value for pathological upgrading in later excisional specimen [5,6].

Papillary lesions of the breast develop as tufts of epithelium with fibrovascular cores that form branching papillae and protrude into ductal lumen. They may present as single or multiple lesions, broad-based or pedunculated. Pathologically, papillary breast lesions range from benign to malignant, including papilloma, papilloma with atypical ductal hyperplasia, papillary carcinoma in situ, microinvasive papillary carcinoma and invasive papillary carcinoma. Intraductal papilloma is the most common papillary lesion of the breast, accounting for about 5% of benign mammary disease. CNB is a well-established diagnostic approach for breast diseases, including papillary lesions. The diagnostic accuracy of CNB depends on characteristics of the lesions, experience of the radiologists and pathologists, the number of the core specimens. Because the pathological nature of a papillary lesion can vary significantly even within a single lesion, insufficient sampling by CNB has long been suspected as a contributing factor for underestimation the severity of the lesion. Actually the reported rates of underestimation in literature vary greatly [7], directly responsible for the lack of consensus for clinical management of nonmalignant papillary breast lesions.

Patient selection, including patient ethnicity, is a potential contributing factor for the reported variability of CNB dignosis upgrading rates. In current study, we retrospectively reviewed our experience with US-CNB in evaluating breast papillary lesions over the past 10 years at a large urban medical center in a primarily African American population. Our results indicate a large percentage (25%) of benign or atypical papillary lesions diagnosed on US-CNB will be upgraded in the final excisional examination in this population.

## Methods

### Patients

Between September 2002 and September 2012, all patients underwent US-CNB of breast lesion in our medical center with diagnosis of papillary lesion (papilloma, papillomatosis, papilloma with atypical ductal hyperplasia, papillary carcinoma in situ, and invasive papillary carcinoma) were enrolled in this study. The primary indication for US-CNB was the sampling and diagnosis of breast nodule/mass detected by palpitation, ultrasound or mammography. All patients with core biopsy were followed for their further clinical management including surgical procedure and radiological examination. This study protocol was reviewed and approved by Newark Beth Israel Medical Center: Institutional Review Board, Institutional Ethics Committee.

### Biopsy procedure

Breast US imaging, using both grayscale and color and power Doppler US, was performed using high-resolution scanners with high-frequency linear-array 10–14-MHz transducers: 15L8W broadband transducer on Acuson, Sequoia (Siemens Medical Solutions, Mountain View, CA), or PLT1204AX matrix transducer on Aplio 80 (Toshiba Medical Systems, Tokyo, Japan). The lesions were first imaged with gray-scale US and then color Doppler US was performed. 14-G and 18-G US-guided core biopsy needles with a spring-loaded core biopsy device (Bard Magnum; Bard Urological, Covington, GA) were used in this study. Two to five core samples per lesion were biopsied.

The US-guided vacuum-assisted biopsies (VABs) were performed using a 10-G handheld VAB system (Vacora; Bard Urological).

Mammography was performed in mediolateral and craniocaudal imaging planes using film-screen mammographic equipment (M-IV mammographic unit; Hologic, Bedford, MA). Additional mammographic projections were performed as needed. The stereotactic guided core biopsies were performed on a digital stereotactic table (LoRad DSM; Hologic) using an 11-G Mammotone directional VAB device (Ethicon Endo-Surgery, Cincinnati, OH).

All static and color Doppler images were stored on the Picture Archiving and Communication System of our medical center.

### Final diagnosis

The recorded pathology results from the percutaneous breast US-CNB and surgical excisional specimens were included for the study and all pathology cases were reviewed by 2 experienced pathologists.

The papillary lesions were categorized based on the core biopsy result into three groups: benign, atypical or malignant. The benign category included the diagnoses of benign papillary lesion, intraductal papilloma, sclerotic intraductal papilloma, intraductal papilloma with florid epithelial hyperplasia without atypia, papillomatosis, and papillary hyperplasia. The atypical category included the diagnoses of papilloma with atypical ductal hyperplasia, papilloma with atypia, atypical papilloma, The malignant category included malignant papillary lesion, papillary ductal carcinoma in situ, intraductal papillary carcinoma, microinvasive and invasive papillary carcinoma. For each papillary lesion, the patient’s medical records were reviewed to identify whether surgery was performed. For surgically excised lesions, the final surgical pathology (gold standard in this study) was classified into benign, atypical or malignant categories and compared with the initial CNB result. Concordance or discordance between the two results was documented. An upgraded lesion was defined as a lesion that was benign (with/without atypia) on CNB but showed (in situ or invasive) malignancy in the excision. It was assumed that the lesions were fully removed at the time of surgery and follow-up imaging was therefore not documented for these lesions.

### Immunohistochemistry

Immunostains were performed following the protocols set up by manufacturer (Leica Microsystem, Buffalo Grove, IL). CK5/6 and CK903 antibodies were pre-made (Leica Bond, Newcastle, UK).

### Statistical methods

Descriptive statistics were calculated for all variables, including means, standard deviations, and proportions. A t-test was used to characterize the relationship between quantitative variables, and the Fisher exact test was used to characterize the relationship between categorical variables. Throughout all analysis, 95% confidence intervals were calculated for all parameters.

## Results and discussion

### Patient demographics

Over the 10 years of our review period, a total of 64 patients were diagnosed to have breast papillary lesion by CNB. Eighty percent (51 patients) of these patients were African-American and the mean age of the patients is 54 years old. The patients’ mean age and follow-up status in the benign, atypical and malignant categories were listed in Table 1.

**Table 1:** Ultrasound fine needle biopsy patient demographics

	**African American(%)**	**Average age(years)**	**Surgical follow-up**	**Radiological follow-up**
Benign	43/55 (78)	51.4	45%	61.3%
Atypical	5/6 (83)	60.8	50%	100%
Malignant	3/3 (100)	79.7	33%	66.7%
Total	51/64 (80)	53.6	45%	67.5%

### Cases with surgical follow-up

Of the twenty-nine CNB patients who were managed by surgical excision within six months after CNB diagnosis, 25 were African-American, 3 Hispanic and 1 Asian American.

**Figure 1:**
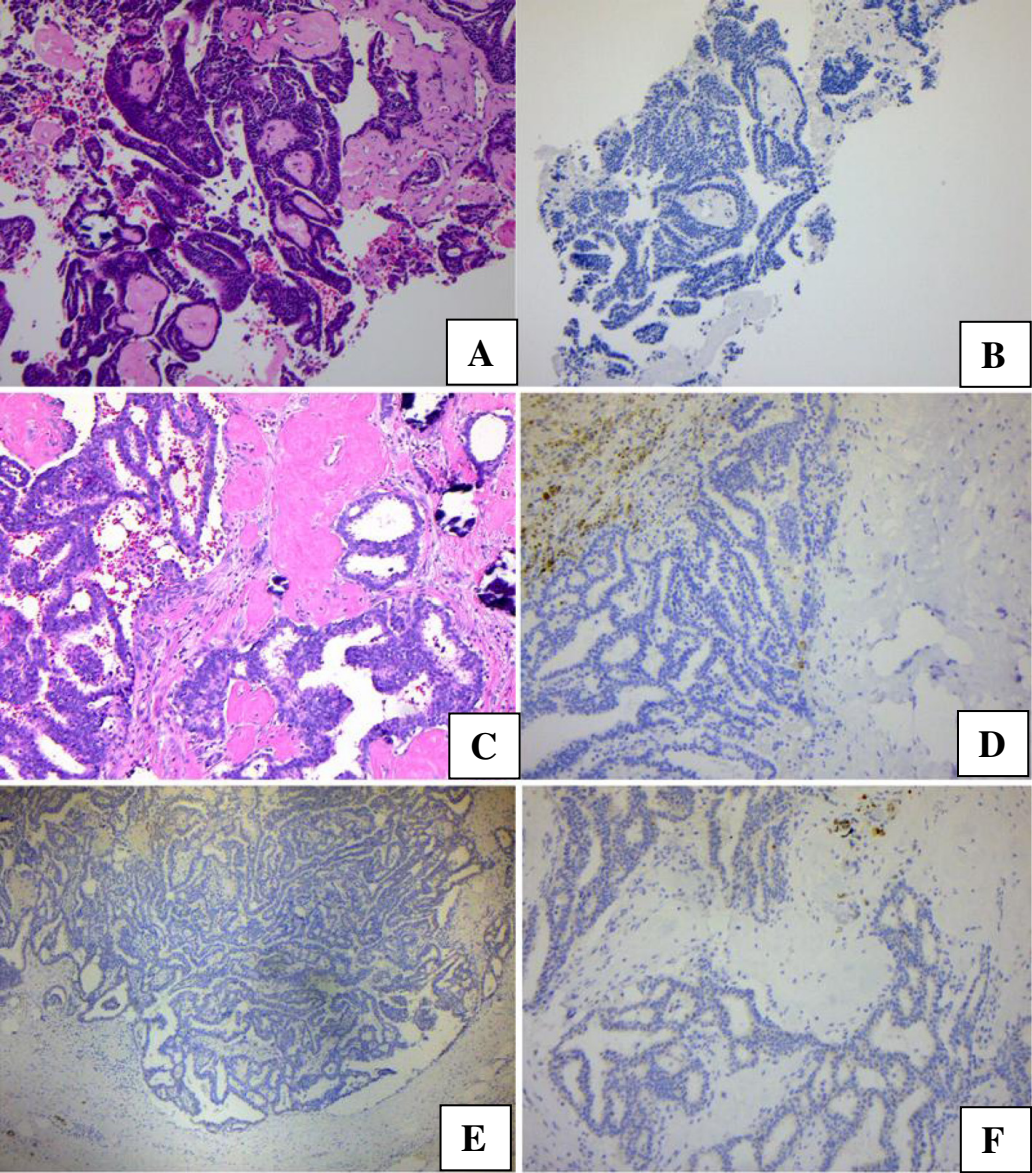
**Core needle biopsy of an originally diagnosed atypical intraductal papilloma (1A, H & E, original magnification: × 100); retrospective CK5/6 immunohistochemical analysis showed minimal positive staining (1B, original magnification × 100).** Mastectomy examination upgraded the lesion to invasive papillary carcinoma (1**C**, **H** &**E**, original magnification: × 100), and absence of CK5/6 stain (1**D**, original magnification × 100), CK903 (1**E**, original magnification × 100), p63 (1**F**, original magnification × 100).

Pathology of the lumpectomy showed: five of the 25 (20%) benign papillary lesions on needle biopsy were upgraded to intraductal or invasive papillary carcinoma; 2 of the 3 atypical papillary lesion cases on core biopsy were upgraded (67%), one into intraductal papillary carcinoma, the other invasive papillary carcinoma; the only case of malignant papillary lesion on core needle biopsy remained as intraductal papillary carcinoma on lumpectomy (Table 2, Figure 1A – F).

**Table 2:** Patients with upgraded lesions on subsequent excision

	**Age**	**Original core needle biopsy diagnosis**	**Immunostain on core needle biopsy**	**Diagnosis after surgical excision**	**Immunostain on surgical excision**	**Operation**
1	42 y	Papilloma	Originally; Minimal CK5/6, CK903stains.	Invasive Ca	No	Lumpectomy
2	74 y	Papilloma	Retrospectively; Minimal CK5/6, CK903 stains	Invasive papillary Ca	No	Mastectomy
3	75 y	Papilloma	Originally; Focal weak CK5/6, CK903 stain.	DCIS	Focal weak CK5/6, CK903 stain	Lumpectomy
4	76 y	Papillomatosis	Retrospectively; Minimal CK5/6, CK903 stains	DCIS, microinvasive	Minimal CK5/6, CK903 stains	Lumpectomy
5	56 y	Papilloma	Retrospectively; Reduced CK5/6, CK903 stains	DCIS	Reduced CK5/6, CK903 stain	Lumpectomy
6	54 y	Papilloma w/atypia	Originally; Reduced CK5/6, CK903 stains	DCIS	Reduced CK5/6, CK903 stains	Lumpectomy
7	75 y	Papilloma w/atypia	Originally; Reduced CK5/6, CK903 stains	Invasive papillary Ca	No	Mastectomy

The average age of the patients who were upgraded by surgical excision was 64.6 years; this was significantly higher than that of the non-upgraded patients (47.7 years old, p = 0.017). Differences between other variables, including lesional size, distance from nipple, presence of microcalcifications, and BI-RADS grade, did not reach statistical significance (Table 3).

**Table 3:** Difference between clinicopathological variables in groups with or without upgrading

	**Non**-**upgraded group**	**Upgraded group**	**P value**
Mean age (years)	47.7 ± 11.1	64.6 ± 13.7	0.017
Lesion size (mm)	16.8 ± 9.1	22.2 ± 12.2	N.S.
Distance from nipple (cm)	3.2 ± 4.1	8.3 ± 4.6	N.S.
Microcalcification	4/14	1/4	N.S.
BI-RADS	4B: 7/11; 4A: 2/11; 2: 2/11; not evaluated: 11	4B: 3/4; 4A: 1/4; not evaluated: 3	N.S.

*N.S*. not significant.

CK5/6 and CK903 immunostains were performed on the CNBs of our No. 7 patient (Table 2) and her malignant follow-up specimens. Upon retrospective review, reduced CK5/6 and CK903 immunostains were helpful for upgrading three of the CNBs from intraductal papilloma into malignancy (DCIS or invasive carcinoma, Illustrated in Figure 1D – E).

## Discussion

To the best of our knowledge, there have not been published studies specifically examining the accuracy of CNB for breast non-malignant papillary lesions in specific ethnic groups. However, clinical and biological differences are known to exist between the ethnic groups. For example, African-American women with breast cancer are reported to be more likely to have advanced disease at diagnosis, estrogen receptor negative, a high S-phase fraction, higher risk of recurrence and poorer prognosis than Caucasian women [8]. Insulin-like growth factor II receptor expression is higher in breast tumor of African-American women than those of any other ethnic group [9]. Compared to Caucasian women, African American women carry more risk factors for breast cancer: younger age at first live birth, less and shorter breast feeding, higher BMI, less moderate physical activity [10]. While alcohol consumption increase breast cancer risk in Caucasian women, recent alcohol consumption apparently does not affect breast cancer risk in African American women, while early age drinking in this ethnic group seems to decrease the risk [11]. Association between 5p12 genomic markers and breast cancer susceptibility was identified in Caucasians and East-Asians, but not in Africans or African-Americans [12]. Results in our study show a large percentage (25%) of benign or atypical papillary lesions diagnosed on CNB will be upgraded in the final excisional examination in a primarily African-American patient population. Our upgraded rate is slightly higher than the median percent of underestimation (16.6%) based on a meta-analysis of 34 studies from mixed ethnic groups [7]. While this is an interesting trend, the small patient number of our study warrants further investigation of this question.

Pathological classification of breast papillary lesions on CNB, especially on H & E preparation alone, can be challenging. Classical intraductal papilloma shows obvious myoepithelial cells covering fibrovascular cores, a polymorphic epithelial cells and normochromatic nuclei; on the other hand, papillary DCIS has no or scant myoepithelial cells, its neoplastic epithelial cells are monomorphic with rigid architecture, and hyperchrmomatic nuclei [13]. Atypical papillary lesion is defined as papilloma with focal population of monotonous cells with the cytological and architectural features of low grade ductal neoplasia, scant or absent myoepithelial cells in these foci, and the atypical epithelial cells usually show lack of staining for high-molecular weight keratin and uniform positivity for estrogen receptor. Over the years, many investigators have suggested immunohistochemical stains on CNB enhance diagnostic accuracy [14-17]. In this sense, it is interesting to note that in a meta-analysis of published studies, a decreasing trend of underestimation was obvious over time between CNB and surgical excision [7]. Furthermore, a recent study from Toronto, Ontario points out that correlation between CNB and excision diagnoses for breast papillary lesions is significantly greater for pathologists specialized in breast than for other pathologists [18]. As part of current study, we performed and re-evaluated immunostains (CK5/6 and CK903) on all seven CNBs that were upgraded to intraductal papillary carcinoma on subsequent specimens. While the discrepancy on some cases was due to sampling, the diagnosis of three core biopsies should be upgraded to malignant according to current diagnostic criteria using immunohistochemistry. Immunostains, as well as other molecular investigative procedures [19], can be very helpful in borderline cases. Another potential source of discrepancy between CNB and excision comes from the heterogeneous nature of the papillary lesion even within a single lesion. Different approaches have been proposed to evaluate lesions of great intrinsic variability, including extended biopsy [20], powered biopsy system [21], and assessment aided by automated intelligent system [22,23]. Application and evaluation of theses novel approaches in breast CNB may help to more accurately classify papillary lesions. When and if possible, more advanced continued education of breast pathology should be encouraged for all practicing pathologists.

Several clinical and radiological variables, including patient’s age, lesion size, distance from nipple, BI-RADS category, have been examined in relations to their predictive value of pathological upgrading from CNB to excisional biopsy. Great efforts have been made, but conclusions from different reports vary significantly [24-27]. In our cohort, the average age of these upgraded cases was 65 years, compared to an average of 48 years among those patients without upgrading. We also evaluated the relationship between upgrading and lesion size, distance from nipple, microcalcification, and BI-RADS category, none of which achieved statistical significance. Hence, in our study of primarily African American population in an urban setting, older age appeared to be an important factor in predicting diagnostic upgrade to a more severe lesion.

## Conclusions

In summary, our study has shown a trend of slightly higher percentage (25%) of upgrading of breast papillary lesions in our predominantly African-American urban population compared to a meta-analysis of mixed ethnic groups published in the literature. The use of immunohistochemistry can increase the diagnostic accuracy of CNB. Early excision of all papillary breast lesions diagnosed by core needle biopsy may be justified in this patient population due to the relatively high chance of a more severe lesion.

## Abbreviations

BI-RADS: Breast Imaging-Reporting and Data System; US-CNB: Ultra-sound guided core needle biopsy; DCIS: Ductal carcinoma in situ; BMI: Body mass index; Ca: Carcinoma.

## Competing interests

The authors declare that they have no competing interests.

## Authors’ contributions

All authors contribute to the design, conduction, data interpretation and writing of this manuscript. All authors read and approved the final manuscript.
